# Left Atrial Appendage Outpouching Mimicking Coronary Artery Aneurysm on Pediatric Echocardiography

**DOI:** 10.1016/j.jaccas.2026.109197

**Published:** 2026-07-09

**Authors:** Michal Huml, Tereza Fremuthová, Jiří Ferda, Josef Sýkora, Jiří Fremuth

**Affiliations:** a Department of Pediatrics, University Hospital in Pilsen and Faculty of Medicine in Pilsen, Charles University, Pilsen, Czech Republic; b Department of Imaging Methods, University Hospital in Pilsen and Faculty of Medicine in Pilsen, Charles University, Pilsen, Czech Republic

**Keywords:** cardiac computed tomography, imaging pitfall, left atrial appendage, multimodality imaging, pediatric echocardiography

## Abstract

**Image(s):**

Multimodality imaging of a tubular left atrial appendage outpouching mimicking coronary artery aneurysm on pediatric echocardiography.

**Case Summary:**

A 3-year-old child with a recent history of febrile illness of unclear etiology underwent transthoracic echocardiography during evaluation of a heart murmur. A tubular outpouching adjacent to the proximal left coronary artery raised concern for possible coronary artery involvement, including coronary artery aneurysm. Doppler findings were nondiagnostic. Cardiac computed tomography demonstrated a narrow connection between the structure and the left atrial appendage. The findings were consistent with a tubular left atrial appendage outpouching without anatomical continuity with the coronary arteries. Three-dimensional reconstruction clarified the projection-dependent overlap responsible for the false echocardiographic appearance.

**Take-Home Messages:**

Anatomical overlap between a tubular left atrial appendage outpouching and proximal coronary arteries may mimic coronary artery aneurysm on pediatric echocardiography. Careful multiplanar assessment and multimodality imaging can clarify the true anatomical relationship when echocardiographic findings remain inconclusive.

## Graphical Abstract

**Figure:**
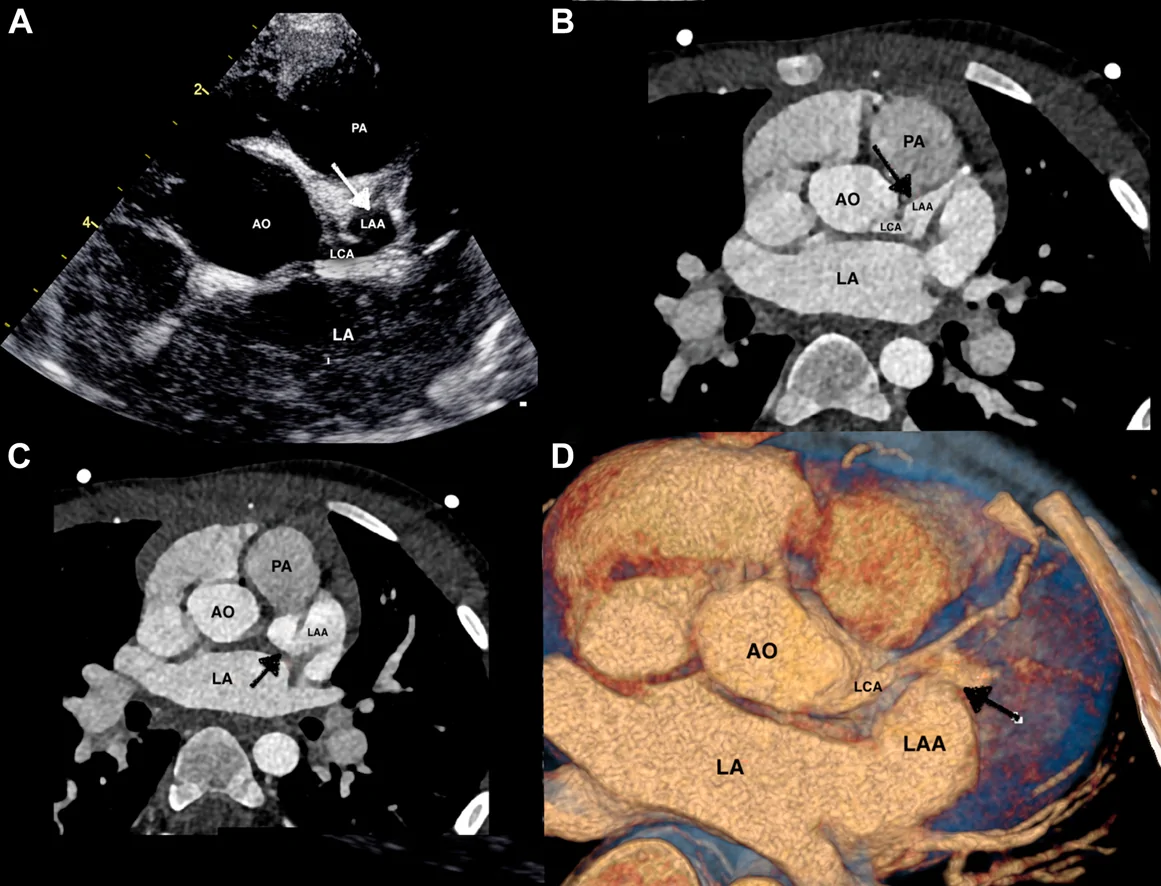

A 3-year-old child with a recent history of febrile illness of unclear etiology underwent transthoracic echocardiography during evaluation of a heart murmur. Transthoracic echocardiography revealed a well-circumscribed tubular structure measuring 4.6 × 4.6 mm with a visible wall located adjacent to the expected course of the left coronary artery (LCA). In a single imaging plane, the structure appeared continuous with the LCA, raising suspicion of coronary artery aneurysm (Figure 1A, Video 1). The combination of prolonged febrile illness and the echocardiographic appearance raised concern for possible coronary artery involvement.

**Figure 1:**
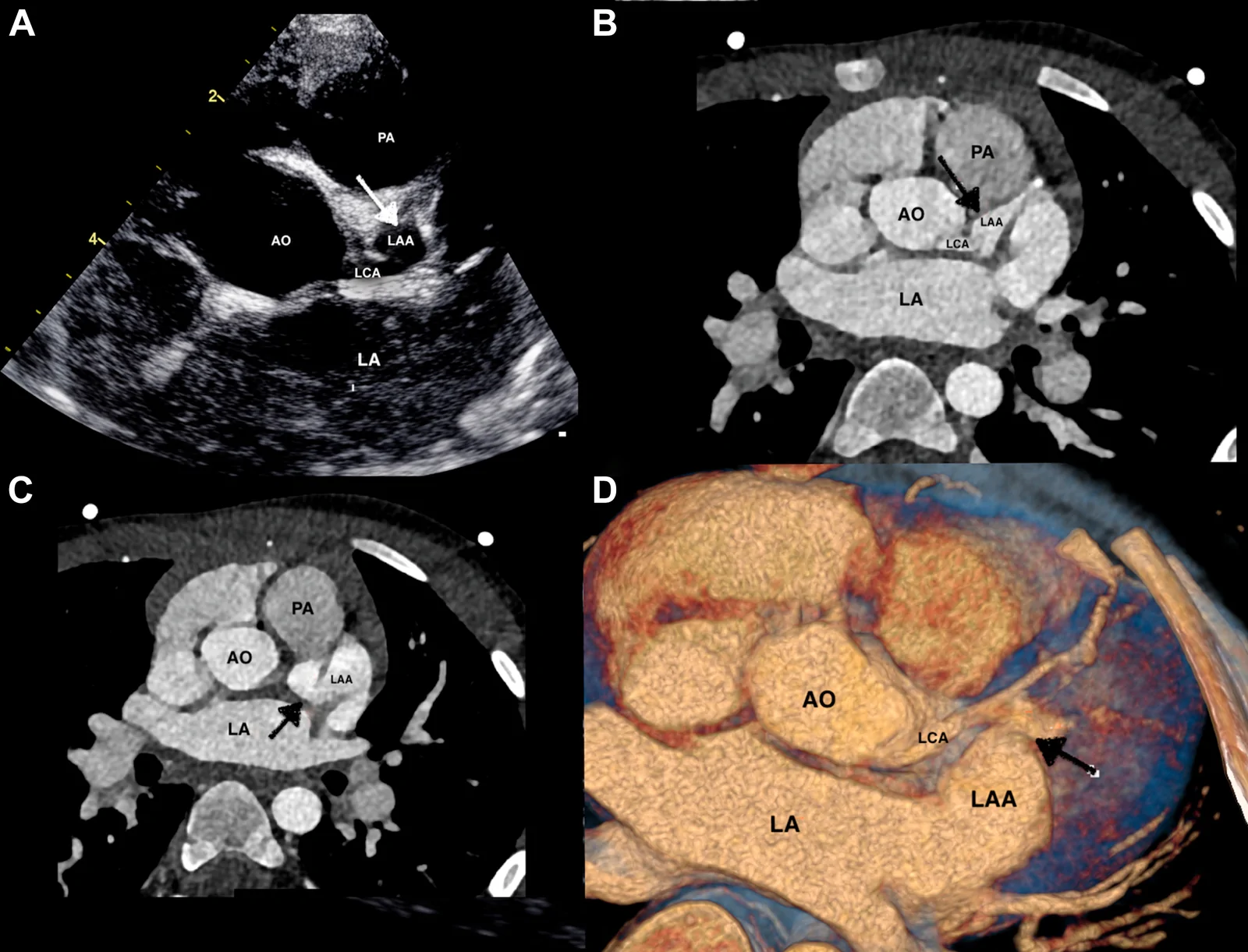
Projection-Dependent Mimicry of Coronary Artery Aneurysm by a Tubular LAA Outpouching (A) Transthoracic echocardiography (parasternal short-axis view) demonstrating a tubular structure (arrow) adjacent to the expected course of the left coronary artery (LCA). In this projection, the structure appears to be in continuity with the proximal LCA, mimicking coronary artery aneurysm. (B) Axial cardiac computed tomography (CT) at a cranial level reproducing the apparent continuity between the tubular outpouching (arrow) and the proximal LCA observed on echocardiography. (C) More caudal CT slice demonstrating a narrow neck (arrow) connecting the tubular outpouching to the left atrial appendage (LAA), consistent with atrial origin and excluding coronary involvement. (D) Three-dimensional CT reconstruction illustrating the spatial relationship between the tubular LAA outpouching (arrow) and the proximal LCA, explaining the false appearance of coronary continuity in this imaging plane. AO = aorta; LA = left atrium; PA = pulmonary artery.

Color Doppler imaging demonstrated low-velocity flow within the structure; however, the flow pattern was nondiagnostic and did not allow reliable differentiation between coronary and atrial origin. Exclusion of coronary artery aneurysm may remain challenging in small children because of projection-dependent overlap and limited spatial resolution. Low-dose cardiac computed tomography (CT) was therefore performed for anatomical clarification.

Cranial CT slices reproduced the apparent continuity between the proximal LCA and a tubular outpouching arising from the left atrial appendage (LAA), explaining the echocardiographic impression (Figure 1B). More caudal slices demonstrated a narrow neck connecting the tubular outpouching to the LAA, with no anatomical continuity with the coronary arteries (Figure 1C). Three-dimensional reconstruction provided intuitive spatial visualization of the relationship between the tubular LAA outpouching and the proximal LCA, explaining the false appearance of coronary continuity (Figure 1D).

LAA outpouchings are increasingly recognized on cardiac CT imaging and may represent clinically silent anatomical variants.^1^ Their close proximity to the proximal coronary arteries may occasionally create diagnostic pitfalls on echocardiography.^2^

LAA flow may demonstrate bidirectional flow patterns, in contrast to the predominantly diastolic forward flow seen in coronary arteries;^3^ however, reliable Doppler differentiation may remain difficult in small children. This case illustrates how anatomical overlap in standard echocardiographic planes may create false suspicion of coronary artery aneurysm and highlights the importance of careful multiplanar assessment before diagnosing coronary abnormalities in pediatric echocardiography.

## Funding Support and Author Disclosures

This work was supported by the Cooperatio Program, Charles University, Prague, Czech Republic (No. 207040). The authors have reported that they have no relationships relevant to the contents of this paper to disclose.

**Take-Home Messages**

• Anatomical overlap between a tubular left atrial appendage outpouching and proximal coronary arteries may mimic coronary artery aneurysm on pediatric echocardiography.
• Careful multiplanar assessment and multimodality imaging can clarify the true anatomical relationship when echocardiographic findings remain inconclusive.

## Appendix

Video 1 Apparent Continuity Between Left Atrial Appendage Outpouching and Proximal Left Coronary Artery Color Doppler echocardiography (parasternal short-axis view) with magnified imaging focused on the proximal left coronary artery, demonstrating dynamic overlap between a tubular left atrial appendage outpouching and the proximal left coronary artery, mimicking coronary artery aneurysm.
